# A Multi-Label Supervised Topic Model Conditioned on Arbitrary Features for Gene Function Prediction

**DOI:** 10.3390/genes10010057

**Published:** 2019-01-17

**Authors:** Lin Liu, Lin Tang, Xin Jin, Wei Zhou

**Affiliations:** 1 School of Information, Yunnan Normal University, Kunming 650500, China; liulinrachel@163.com; 2 Key Laboratory of Educational Informatization for Nationalities Ministry of Education, Yunnan Normal University, Kunming 650500, China; 3 School of Software, Yunnan University, Kunming 650091, China; xinxin_jin@163.com

**Keywords:** multi-label classification, topic model, gene function, probability distribution, Dirichlet-multinomial Regression

## Abstract

With the continuous accumulation of biological data, more and more machine learning algorithms have been introduced into the field of gene function prediction, which has great significance in decoding the secret of life. Recently, a multi-label supervised topic model named labeled latent Dirichlet allocation (LLDA) has been applied to gene function prediction, and obtained more accurate and explainable predictions than conventional methods. Nonetheless, the LLDA model is only able to construct a bag of amino acid words as a classification feature, and does not support any other features, such as hydrophobicity, which has a profound impact on gene function. To achieve more accurate probabilistic modeling of gene function, we propose a multi-label supervised topic model conditioned on arbitrary features, named Dirichlet multinomial regression LLDA (DMR-LLDA), for introducing multiple types of features into the process of topic modeling. Based on DMR framework, DMR-LLDA applies an exponential a priori construction, previously with weighted features, on the hyper-parameters of gene-topic distribution, so as to reflect the effects of extra features on function probability distribution. In the five-fold cross validation experiment of a yeast datasets, DMR-LLDA outperforms the compared model significantly. All of these experiments demonstrate the effectiveness and potential value of DMR-LLDA for predicting gene function.

## 1. Introduction

As the main component of a cell, proteins are the most essential and versatile material of life. Thus, the research on protein functions is of great importance for the development of new drugs, better crops, and the development of synthetic biochemical [1]. In recent years, new protein function prediction methods using machine learning algorithms have proliferated, based on various known information about proteins, and have increasingly become important long-standing research works in the post-genomic era. From the point of molecular biology, a protein is the product of a gene after the process of transcribing, translating, and post-translational modifying. Even though the real function of a gene is to encode one or more proteins executing practical functions, the function of a gene product has usually been regarded as the native function of the gene in gene-level experiments. Therefore, we do not distinguish between gene function and protein function in this paper, which are known collectively as gene function.

The most common computational approach for gene function prediction is to transfer the gene function into some specific features from their sequence or structure similarity, such as BLAST [2]. In addition to sequence similarity, many gene function prediction methods have been exploited in recent years as the additional information extracted from proteins, such as protein structure [3], protein motif, biophysical properties [4], and integrated heterogeneous data sources [5]. In reference [3], Evangelia et al. extract novel shape features from protein structures in the form of local (per amino acid) distribution of angles and amino acid distances, respectively. Each of the multi-channel feature maps is introduced into a deep convolutional neural network (CNN) for function prediction, and the outputs are fused through support vector machines or a correlation-based *k*-nearest neighbor classifier. In addition, automatic prediction using protein–protein similarity information can be further supplemented by experimental data [6,7]; this kind of method assumes that the closely related proteins (or genes) share similar functional annotations on the basis of network structure information. Researchers have made the relevant literature reviews of computational methods on gene function prediction in references [8,9,10].

From the point of machine learning algorithms, predicting gene function based on various data sources is a problem of classification in nature. A gene can be viewed as an instance to be classified—various kinds of data sources (such as an amino acid sequence, textual repositories, and motifs) can be organized into a feature space, so that each gene is represented as a set of attribute values; a function (such as a gene ontology (GO) term [11]) is regarded as a label. As a gene is always annotated by several functions, gene function prediction is actually a process of multi-label classification: a multi-label classifier is trained firstly on constructed attribute features and annotated genes, and then is used to predict function annotations for unannotated genes. From the above analysis, we believe that many multi-label classification algorithms have great potential to predict gene function, such as a support vector machine (SVM), neural network, and decision tree. In reference [12], Celine Vens et al. proposed three multi-label classifiers based on a hierarchical decision tree, and the experimental results from 24 datasets show that these classifiers are powerful and effective for gene function prediction.

In addition to traditional machine learning algorithms, a topic model is a kind of probabilistic generative model that has been applied into gene function prediction. In reference [13], Liu et al. introduced a typical multi-label supervised topic model into gene function prediction, which was called labeled latent Dirichlet allocation (LLDA) and is proposed in reference [14] for text mining. This research is the first effort to apply a multi-label supervised topic model into gene function prediction. Compared with traditional multi-label classification models, LLDA can model a function label as a topic, and thus can not only work out the function probability distributions over gene instances effectively, but can also directly provide the word probability distributions over functions. Nonetheless, the direct application of LLDA on a gene function dataset can only utilize protein sequence data by formalizing the sequences into a bag of words (BoW), and then the constructed bag of words is used for topic modeling. In other words, due to the restrictions of BoW construction in topic modeling, the feature space was constructed on sequence data rather than multiple biological data. However, we can see from the above paragraph that there are various protein features, such as hydrophobicity and the polarity of amino acids, which have a profound impact on gene structure and function. Apparently, the introduction of multiple kinds of gene features in a multi-label supervised topic model can improve the accuracy of gene function prediction.

Inspired by the application of a multi-label topic model in gene function prediction and a topic model conditioned on arbitrary features named the Dirichlet multinomial regression latent Dirichlet allocation (DMR-LDA) [15], we propose a DMR-LLDA model, which introduces a DMR framework into an LLDA model. Firstly, we describe DMR-LLDA for gene function prediction problem formulation. Then the generative process and the inference algorithm of DMR-LLDA are described. This model is fully compatible with both discrete and continuous features, whose inference is relatively simple. In a five-fold cross validation experiment on verified gene function prediction, DMR-LLDA significantly outperformed LLDA. In addition, the impact of feature variables on prior parameters and the comparison between two kinds of inference algorithms are shown in experimental data. All these experimental results demonstrate the effectiveness and potential value of DMR-LLDA for predicting gene function.

## 2. Methods

### 2.1. Related Definitions and Notations

**Figure 1:**
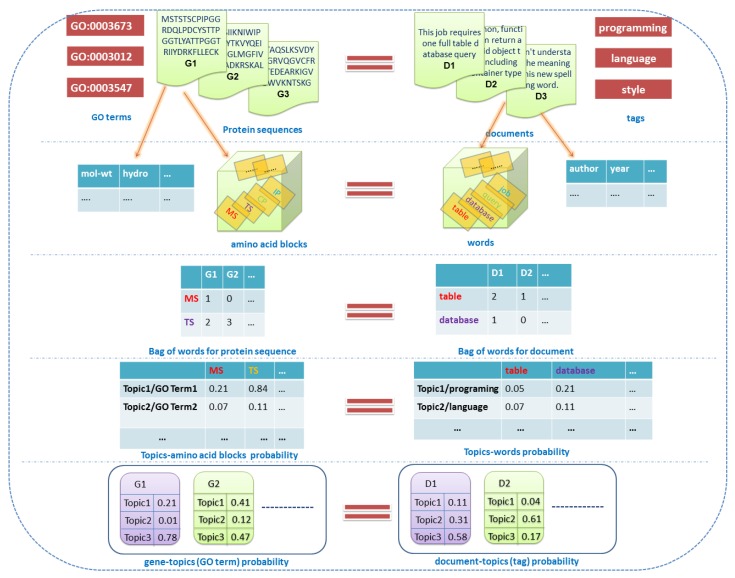
The relationship between protein function prediction and text topic modeling. IP, CP, TS, MS and so on, represent ‘words’, each of which is composed by two amino acid alphabets. Each GO term is started by ‘GO:’.

In this paper, the topic modeling method of gene function prediction reported by reference [13] is utilized. We consider each gene to be a document [16], and GO terms (topics) are shared by a document collection. Meanwhile, we view the extra gene features, except for the bag of amino acid words, as the metadata, such as authors and dates of documents. Therefore, the introduction of extra gene features into topic modeling is similar to introducing metadata into the topic modeling of documents, and the type of metadata may be discrete or continuous. To better understand the practical application of our method, the relationship of text topic modeling and gene function predicting is illustrated by Figure 1.

In Figure 1, the right part describes the topic modeling concept of text data, and the left part describes the related concept of gene function data. For all topic models, there are three key concepts: “documents”, “words”, and “topics”. In addition, the supervised topic model introduces “labels” for each document, and the proposed DMR-LLDA model introduces “features” for each document. Therefore, these concepts can now be reformulated with more detail, as follows.

#### 2.1.1. Documents

For text data (right part of Figure 1), document collection is composed of several documents numbered D1 to Dn. In the other side (left part of Figure 1), the gene dataset is composed of several protein sequences, numbered G1 to Gn. Therefore, a document is equivalent to a gene in our model. We suppose that there are D genes in a gene set, which compose the gene space D={1,…,D}, and the gene sample set X including D genes can be represented as $X={\left\{X_{d}\right\}}_{d=1}^{D}$, and $X_{d}$ denotes a gene sample.

#### 2.1.2. Labels

For text data (right part of Figure 1), each document is labeled by one or more tags, such as “programming” and “language”. On the other side (left part of Figure 1), each gene is annotated by several GO terms, such as “GO:0003012” and “GO:0003547”. Therefore, a document tag is equivalent to a GO term in our model, and all of them are called “labels”. In this paper, the gene function label space is expressed as L={1,…,L}. Meanwhile, the observed labels of each gene are described by a sparse binary vector $\Lambda_{d}={\left\{\Lambda_{dl}\right\}}_{l=1}^{L}$, which is defined as follows:

(1) $$\Lambda_{dl}=\left\{ \begin{array}{l} 1,\;l\in L_{d}\\ 0,\;l\notin L_{d} \end{array}\right.$$

where, $L_{d}$ represents the label sub-space of gene $X_{d}$: $L_{d}\subseteq L$.

#### 2.1.3. Words

For text data (right part of Figure 1), word terms are the main component of a document, such as the words “table” and “database”. On the other side (left part of Figure 1), we consider a protein sequence to be a text string, which is defined by a fixed 20 amino acid alphabet (G,A,V,L,I,F,P, Y,S,C,M,N,Q,T,D,E,K,R,H,W). Correspondingly, amino acid blocks are the main components of a protein sequence, which is composed by two or more amino acid alphabets, such as “MS” and “TS”. Therefore, a word term is equivalent to an amino acid block in our model, and all of them are called “words”. Meanwhile, all of the words constitute a vocabulary. In this paper, the amino acid words space is represented as W={1,…,W}. For a gene $X_{d}$, $X_{d}={\left\{x_{dn}\right\}}_{n=1}^{N_{d}}$ denotes that the dth gene is composed by $N_{d}$ observed word samples, and $x_{dn}$ is one of word samples.

#### 2.1.4. Topics

For text data (right part of Figure 1) and gene function data (left part of Figure 1), a “topic” is viewed as a probability distribution over a fixed vocabulary. Taking the text data as an example, the probabilities of the word “table” over “topic 1” are 0.05. For the gene function data, the probabilities of amino acid block MS over “topic 1” are 0.21. Obviously, topics are latent and needed to be inferred by topic modeling. In this paper, the global topic space includes T topics, which is represented as T={1,…,T}. According to the definition of an LLDA model, there is a one-to-one correspondence between label and topic—therefore, L≜T (≜ represents equivalent relationship between two space), T=|T|=L=|L|.

#### 2.1.5. Features

For text data (right part of Figure 1), the metadata of a document can be viewed as document features, such as the tags “author” and “publish year of document”. On the other side (left part of Figure 1), each gene has several extra features, except for its sequence string, such as molecular weight and hydrophobicity. Therefore, the metadata of a document tag is equivalent to an extra feature of the gene in our model, and all of them are called “features”. In this paper, the feature space composed by gene features is expressed as F={1,…,F}. Therefore, there is a set of observed features for gene $X_{d}$, which can be represented as a feature vector: $y_{d}={\left\{y_{df}\right\}}_{f=1}^{F}$.

#### 2.1.6. Others

In addition to the above five concepts, there are three other concepts illustrated in Figure 1. Firstly, the BoW, which is a word–document matrix and the input of the topic model. In an instance in the right part of Figure 1, the word “table” appears two times in document D1. Likewise, the word “MS” appears one time in gene G1. In other words, the element of the BoW represents the times of each word in each document. Meanwhile, there are two probability matrices that appear in Figure 1: one is the topic (label)–word probability matrix, and the other is the document (gene)–topic probability matrix. All of them are represented as parameter vectors for each topic or gene in the topic model.

A topic corresponds to a multinomial distribution of word space W, whose parameter vector is $\theta_{t}={\left\{\theta_{tw}\right\}}_{w=1}^{W}$, and $\theta_{tw}$ is the probability of word w under topic t; a gene $X_{d}$ corresponds to a multinomial distribution of the topics space T, whose parameter vector is $\pi_{d}={\left\{\pi_{dt}\right\}}_{t=1}^{T}$, and $\pi_{dt}$ is the topic weight of topic t under gene $X_{d}$. Finally, we utilize a feature parameter vector $\beta_{t}={\left\{\beta_{tf}\right\}}_{f=1}^{F}$ to represent the relationship between features (*f*) and topics (*t*) in making features influence the choice of topic.

Note that the shared parameters of a whole gene set, such as topic–word parameter θ, are called “global parameters” in this paper. Correspondingly, the parameter of one gene is called a local parameter, such as gene–topic (label) parameter π.

### 2.2. Overview of the Dirichlet–Multinomial Regression Latent Dirichlet Allocation Topic Modeling Process

Based on the above notation, we can provide the description of a gene function dataset as follows.

The gene $X_{d}$ is composed of $N_{d}$, which are observed samples, and the word index of each sample $x_{dn}$ comes from the vocabulary $w_{dn}\in W_{d}$. Thus, the gene can also be represented as $W_{d}={\left\{w_{dn}\right\}}_{n=1}^{N_{d}}$, where $W_{d}$ is the local word subspace of $X_{d}$. In addition, the latent variables of gene $X_{d}$ is its topic subset $T_{d}={\left\{t_{dn}\right\}}_{n=1}^{N_{d}}$, where $t_{dn}\in T_{d}$, and $T_{d}$ is the local topic subspace, $W_{d}\subseteq W$, and $T_{d}\subseteq T$. Specifically, each gene shares the global topic space $T_{d}\equiv T$, where d∈D. In this case, we suppose that each word $w\in W_{d}$ of each gene $X_{d}$ shares the same feature vector: $y_{dw}\equiv y_{d}={\left\{y_{df}\right\}}_{f=1}^{F}$.

Then, the topic modeling process of our model can be interpreted as follows: for the training set, learning the unknown parameter $\theta_{t}$, $\pi_{d}$, and $\beta_{t}$ from the observed variables $W_{d}$, $T_{d}$, and $y_{d}$; for the testing set, predicting $T_{d}$ and $\pi_{d}$ from known parameters $\theta_{t}$ and $\beta_{t}$, and the observed variables $W_{d}$ and $y_{d}$. Obviously, $\theta_{t}$ and $\beta_{t}$ are global parameters, which are shared by the whole dataset. The above two steps are also called model training and predicting, and are realized by learning and inference algorithms, such as Gibbs sampling [17] and variable inference [18].

**Figure 2:**
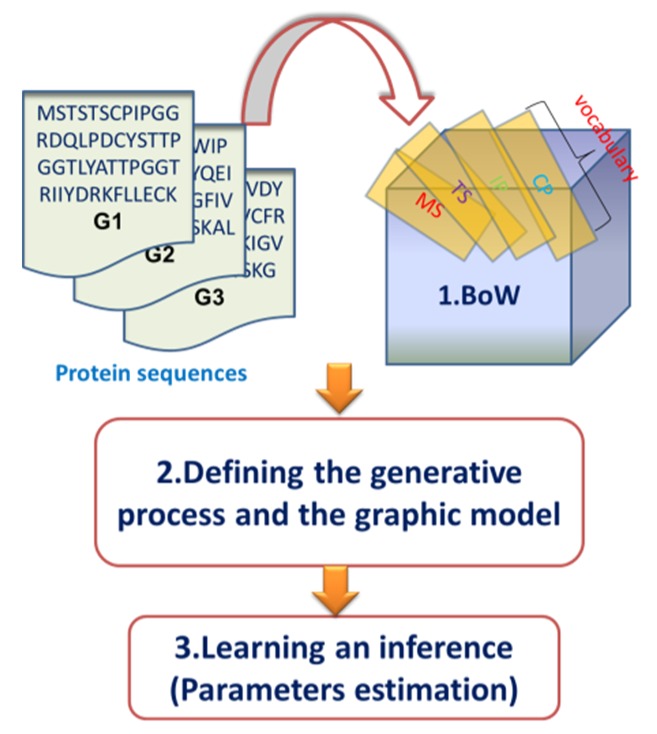
An overview of the topic modeling process.

Moreover, there are two steps before model training and predicting: BoW construction and model description. Since we constructed the BoW of the gene in exactly the same way as reference [13], this step will be not repeated in this paper. For model description, there are usually two ways to describe a probabilistic graphical model, including the generative process and the graphic model, which are discussed in the next sections. The overview of our topic modeling process is depicted in Figure 2.

### 2.3. Description of the Dirichlet–Multinomial Regression Latent Dirichlet Allocation Model

This section provides the description of DMR-LLDA, including its generative process and graphic model. It is worth noting that our DMR-LLDA introduces the DMR framework for gene features based on the LLDA model, so this paper emphasizes the DMR part rather than the classic LLDA.

According to DMR framework, each word sample $x_{dn}$ of gene $X_{d}$ is a “individual”, and all of the samples ${\left\{x_{dn}\right\}}_{n=1}^{N_{d}}$ are divided into $\left|W_{d}\right|=W_{d}$ groups by word number $\left|W_{d}\right|=W_{d}$. A bag of words ${\left\{x_{dn}\right\}}_{n=1}^{N_{dw}}$ is composed by $N_{dw}$(the number of word w appeared in gene $X_{d}$) samples of the w-th group, and corresponds to a feature vector $y_{dw}$, which influences the latent topic t∈T choice of all samples.

We suppose that

(2) $${\tilde{\alpha }}_{dwt}=\exp\left(y_{dw}\beta_{t}^{T}\right)\equiv \exp\left(y_{d}\beta_{t}^{T}\right)={\tilde{\alpha }}_{dt},\;w\in W_{d}$$

In Equation (2), $\beta_{t}={\left\{\beta_{tf}\right\}}_{f=1}^{F}$ represents feature parameters that correspond to topic t. Likewise, each bag of words w of gene $X_{d}$ shares the same clustering random variable:

(3) $$\exp\left(\xi_{dwt}\right)\equiv \exp\left(\xi_{dt}\right)=\zeta_{dt},\;w\in W_{d}$$

where $\pi_{dt}$ is the selecting probability of the *n*-th word sample of gene $X_{d}$, which chooses topic t and maximizes the utility selection $U_{dnt}$. In addition, $\pi_{d}={\left\{\pi_{dt}\right\}}_{t=1}^{T}$ is the topic weight vector of gene $X_{d}$, which obeys the Dirichlet distribution of parameter ${\left\{\delta_{d}^{-1}{\tilde{\alpha }}_{dt}\right\}}_{t=1}^{T}$:

(4) $$p\left(\pi_{d}\right)=p\left({\left\{\pi_{dt}\right\}}_{t=1}^{T}\right)=\frac{\Gamma \left(\sum_{t=1}^{T}\delta_{d}^{-1}{\tilde{\alpha }}_{dt}\right)}{\prod_{t=1}^{T}\Gamma \left(\delta_{d}^{-1}{\tilde{\alpha }}_{dt}\right)}\prod_{t=1}^{T}\pi_{dt}^{\delta_{d}^{-1}{\tilde{\alpha }}_{dt}-1}=\frac{\Gamma \left(\sum_{t=1}^{T}\alpha_{dt}\right)}{\prod_{t=1}^{T}\Gamma \left(\alpha_{dt}\right)}\prod_{t=1}^{T}\pi_{dt}^{\alpha_{dt}-1}$$

where $\alpha_{dt}$ is the hyper-parameter of $\pi_{dt}$:

(5) $$\alpha_{dt}=\delta_{d}^{-1}{\tilde{\alpha }}_{dt}=\delta_{d}^{-1}\exp\left(y_{d}\beta_{t}^{T}\right)$$

The description of DMR-LLDA from the global and local perspective is shown below.

From the global perspective, each topic t∈T can be represented as a multinomial distribution over vocabulary W, whose parameter is expressed as vector $\theta_{t}={\left\{\theta_{tw}\right\}}_{w=1}^{W}$, and we suppose that $\theta_{t}$ obeys Dirichlet conjugate prior distribution. Each topic t∈T corresponds to a feature weight parameter vector $\beta_{t}$, which obeys the normal distribution of parameter $\left(\mu ,\sigma^{2}\right)$.

From the local perspective, each gene $X_{d}$ is composed by $N_{d}$ observed samples, which corresponds to local word number subset $W_{d}={\left\{w_{dn}\right\}}_{n=1}^{N_{d}}$ and local latent topic number subset $T_{d}={\left\{t_{dn}\right\}}_{n=1}^{N_{d}}$, where $T_{d}$ obeys multinomial distribution of parameter $\pi_{d}$. The local observed word subspace of gene $X_{d}$ is $W_{d}$, the local observed label subspace is $L_{d}$, and the local observed feature subspace is $F_{d}$. Each label $l\in L_{d}$ corresponds to a topic t∈T, where $T_{d}\equiv L_{d}\subseteq T$ and $\Lambda_{d}={\left\{\Lambda_{dt}\right\}}_{t=1}^{T}={\left\{\Lambda_{dl}\right\}}_{l=1}^{L}$. The dimension of topic weight $\pi_{d}={\left\{\pi_{dt}\right\}}_{t\in T_{d}}$ corresponds to $T_{d}$, which is $T_{d}=|T_{d}|=|L_{d}|\neq T$. At the same time, the range of topics on feature weight parameter vector $\beta_{t}$ is limited to $t\in T_{d}$. In addition, $y_{d}\beta_{t}^{T}$ decides the hyper-parameter $\alpha_{d}={\left\{\alpha_{dt}\right\}}_{t\in T_{d}}={\left\{\alpha_{dt}\right\}}_{t\in L_{d}}$ of $\pi_{d}$, which is the dot-product of feature vector $y_{d}$, corresponding to feature subspace $F_{d}$ and its weighted parameter vector $\beta_{t}$.

Above all, the Dirichlet prior hyper-parameter $\alpha_{d}$ of $\pi_{d}$ can be expressed as

(6) $$\alpha_{d}={\left\{\alpha_{dl}\right\}}_{l\in L_{d}}={\left\{\alpha_{dt}\Lambda_{dt}\right\}}_{t=1}^{T},\;\left|\alpha_{d}\right|=T_{d}=L_{d}$$

where $\alpha_{dt}$ is computed by Equation (5). The local topic weight $\pi_{d}$ can be also represented as

(7) $$\pi_{d}={\left\{\pi_{dt}\right\}}_{t\in T_{d}}={\left\{\pi_{dt}\Lambda_{dt}\right\}}_{t=1}^{T},\;\left|\pi_{d}\right|=T_{d}=L_{d}$$

**Figure 3:**
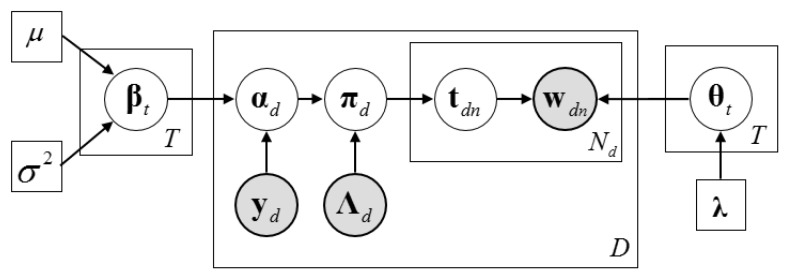
The graphic model of Dirichlet multinomial regression latent Dirichlet allocation (DMR-LLDA).

Given the above, the generative process of DMR-LLDA can be described as follows. The corresponding graphical model is shown in Figure 3.

For each global topic t∈T={1,…,T}, we can

(a) Generate a feature weighted parameter vector $\beta_{t}={\left\{\beta_{tf}\right\}}_{f=1}^{F}$ of topic t from F dimension’s normal distribution of parameter $\left(\mu ,\sigma^{2}\right)$:

(8) $$\beta_{t}={\left\{\beta_{tf}\right\}}_{f=1}^{F}∼N\left(\mu ,\sigma^{2}I\right)$$

(b) Generate a multinomial parameter vector $\theta_{t}={\left\{\theta_{tw}\right\}}_{w=1}^{W}$ from a W dimension Dirichlet distribution:

(9) $$\theta_{t}={\left\{\theta_{tw}\right\}}_{w=1}^{W}∼\mathrm{Dir}\left(\lambda \right)$$

For each gene $X_{d}$, d∈D={1,…,D}. This means that

(a) We suppose that $\alpha_{dt}=\delta_{d}^{-1}{\tilde{\alpha }}_{dt}$ ($\delta_{d}>0$) as the Dirichlet prior hyper-parameter of the topic weight

(10) $$\alpha_{dt}=\delta_{d}^{-1}\exp\left(y_{d}\beta_{t}^{T}\right)=\delta_{d}^{-1}\exp\left(\sum_{f=1}^{F}y_{df}\beta_{tf}\right)$$

(b) The binary vector $\Lambda_{d}={\left\{\Lambda_{dt}\right\}}_{t=1}^{T}$ limits the prior hyper-parameter $\alpha_{d}$ of local topic weight

(11) $$\alpha_{d}\Lambda_{d}={\left\{\alpha_{dt}\Lambda_{dt}\right\}}_{t=1}^{T}$$

(c) We can generate local weight topic vector of topic t from a Dirichlet distribution:

(12) $$\pi_{d}={\left\{\pi_{dt}\Lambda_{dt}\right\}}_{t=1}^{T}∼\mathrm{Dir}\left(\alpha_{d}\Lambda_{d}\right)$$

(d) For each word sample $x_{dn}$, we can

i. Generate topic number $t_{dn}$ of $x_{dn}$ from T dimensions’ multinomial distribution of parameter $\pi_{d}$:

(13) $$t_{dn}∼\pi_{d}\;\mathrm{or}\;T_{d}={\left\{t_{dn}\right\}}_{n=1}^{N_{d}}∼\mathrm{Mul}\left(\pi_{d},N_{d}\right)$$

ii. Generate word number $w_{dn}$ of $x_{dn}$ from W dimensions’ multinomial distribution of parameter $\theta_{t_{dn}}$:

(14) $$w_{dn}∼\theta_{t_{dn}}\;\mathrm{or}\;W_{d}={\left\{w_{dn}\right\}}_{n=1}^{N_{d}}∼\mathrm{Mul}\left(\theta_{t_{dn}},N_{t_{dn}}\right)$$

As we can see from Figure 3, $\alpha_{d}$ is computed by feature vector $y_{d}$ and its weighted parameter. Therefore, $\alpha_{d}$ is a parameter rather than a random variable in the LLDA.

In our DMR-LLDA model, the unknown parameters to be estimated are the global feature parameter β, the global topic–word multinomial distribution parameter θ, and the local topic weight π. The hidden variable to be estimated is T. The known data are the observed word samples W and binary vector Λ. The joint distribution of (β,π,θ,T,W) is shown in Equation (15):

(15) $$\begin{array}{l} \;p\left(\beta ,\pi ,\theta ,T,W\;|\mu ,\sigma^{2},\Lambda ,\lambda \right)\\ =p\left(\beta |\mu ,\sigma^{2}\right)\cdot \prod_{t=1}^{T}p\left(\theta_{t}|\lambda \right)\cdot \prod_{d=1}^{D}p\left(\pi_{d}|\Lambda_{d},\alpha_{d}\right)\prod_{n=1}^{N_{d}}\;p\left(t_{dn}|\pi_{d}\right)p\left(w_{dn}|t_{dn},\theta \right) \end{array}$$

Above all, the proposed method utilizes extra features as the prior knowledge of the related distribution, which is able to gain more reliable prior distribution for the LLDA; then a more precise estimation of posterior distributions is obtained.

### 2.4. Inference Algorithm of Dirichlet–Multinomial Regression Latent Dirichlet Allocation

The core learning task of DMR-LLDA is to compute the parameters (π,θ,β) and posterior distribution p(π,θ,β,T |W). The posterior estimation represents the estimating value of the parameter under the training set. The prediction process of DMR-LLDA is that on the basis of the estimated three parameters and a hidden variable, we update the unknown local parameter π and hidden variable of the test gene by fixing the learned global parameters β and θ; then, we get the corresponding relationship between the label and the topic. The Gibbs sampling algorithm and the variable Bayesian algorithm are two essentially approximate inference algorithms of a probabilistic graphic model, and the purpose of them is universal. In order to compare their impact on the model performance of difference inference algorithms, we designed a collapsed Gibbs sampling algorithm (CGS), a collapsed variable Bayesian algorithm (CVB), and a zero-order variational Bayesian algorithm (CVB0) for DMR-LLDA, with detail as follows.

#### 2.4.1. The Collapsed Construction of Dirichlet–Multinomial Regression Latent Dirichlet Allocation

First of all, after the integration of model parameters (π,θ) in a joint distribution, a semi-collapsed (β,T,W) joint distribution is obtained:

(16) $$\begin{array}{l} p\left(\beta ,T,W|\Lambda ,\alpha ,\lambda ,\mu ,\sigma^{2}\right)=p\left(\beta |\mu ,\sigma^{2}\right)p\left(T|\Lambda ,\alpha \right)p\left(W|T,\lambda \right)\\ \propto \prod_{t=1}^{T}\prod_{f=1}^{F}\frac{1}{\sqrt{2\pi \sigma^{2}}}e^{-\frac{{\left(\beta_{tf}-\mu \right)}^{2}}{2\sigma^{2}}}\cdot \prod_{d=1}^{D}\frac{\Gamma \left(\sum_{t=1}^{T}\alpha_{dt}\Lambda_{dt}\right)}{\Gamma \left(\sum_{t=1}^{T}\alpha_{dt}\Lambda_{dt}+N_{d}\right)}\prod_{t=1}^{T}\frac{\Gamma \left(\alpha_{dt}\Lambda_{dt}+N_{dt}\Lambda_{dt}\right)}{\Gamma \left(\alpha_{dt}\Lambda_{dt}\right)}\\ \cdot \prod_{t=1}^{T}\frac{\Gamma \left(\sum_{w=1}^{W}\lambda_{w}\right)}{\Gamma \left(\sum_{w=1}^{W}\lambda_{w}+N_{t}\right)}\prod_{w=1}^{W}\frac{\Gamma \left(\lambda_{w}+N_{tw}\right)}{\Gamma \left(\lambda_{w}\right)} \end{array}$$

The predictive probability distribution for the topic assignment of sample $x_{dn}$ is

(17) $$\begin{array}{l} p\left(t_{dn}=t|T^{\left(\backslash dn\right)},W^{\left(\backslash dn\right)},\Lambda ,\alpha ,\lambda \right)\\ \propto p\left(t_{dn}=t,w_{dn}=w|T^{\left(\backslash dn\right)},W^{\left(\backslash dn\right)},\Lambda ,\alpha ,\lambda \right)\\ =p\left(t_{dn}=t|T^{\left(\backslash dn\right)},\Lambda ,\alpha \right)p\left(w_{dn}=w|t_{dn}=t,T^{\left(\backslash dn\right)},W^{\left(\backslash dn\right)},\lambda \right)\\ \propto \left(\alpha_{dt}+N_{dt}^{\left(\backslash dn\right)}\right)\Lambda_{dt}\frac{\lambda_{w}+N_{tw}^{\left(\backslash dn\right)}}{\sum_{w=1}^{W}\lambda_{w}+N_{t}^{\left(\backslash dn\right)}} \end{array}$$

$N_{dt}^{\left(\backslash dn\right)}\Lambda_{dt}$ is the number of samples that are assigned to the corresponding topic t of gene $X_{d}$, except for sample $x_{dn}$. $N_{tw}^{\left(\backslash dn\right)}$ is the number of samples that are assigned to the word w of topic t, except for sample $x_{dn}$; therefore, $N_{t}^{\left(\backslash dn\right)}=\sum_{w=1}^{W}N_{tw}^{\left(\backslash dn\right)}$.

In Equation (17), $\alpha_{dt}$ is optimized by local observed feature vector $y_{d}={\left\{y_{df}\right\}}_{f=1}^{F}$ and global feature parameter $\beta_{t}={\left\{\beta_{tf}\right\}}_{f=1}^{F}$, whose updating equation is Equation (5). To simplify the updating equation, we first suppose that $\log\delta_{d}^{-1}=y_{df_{default}}\beta_{tf_{default}}$, and then an item of hidden global feature parameter $\beta_{tf_{default}}$ is added for global feature parameter $\beta_{t}={\left\{\beta_{tf}\right\}}_{f=1}^{F}$, which corresponds to a “fake” observed feature $y_{dF_{default}}=1$. Thus, the updating equation of $\alpha_{dt}$ is

(18) $$\alpha_{dt}^{new}=\exp\left({\hat{y}}_{d}{\hat{\beta }}_{t}^{T}\right)=\exp\left(y_{df_{default}}\beta_{tf_{default}}+\sum_{f=1}^{F}y_{df}\beta_{tf}\right)$$

(19) $$\begin{array}{l} {\hat{\beta }}_{t}=\left\{\beta_{t},\beta_{tF_{default}}\right\}=\left\{\beta_{t1},\beta_{t2},\ldots ,\beta_{tF},\beta_{tF_{default}}\right\}\\ {\hat{y}}_{d}=\left\{y_{d},y_{dF_{default}}\right\}=\left\{y_{d1},y_{d2},\ldots ,y_{dF},1\right\} \end{array}$$

#### 2.4.2. The Optimization of the Feature Parameters of Dirichlet–Multinomial Regression Latent Dirichlet Allocation

For Gibbs sampling or variable Bayesian, we need to update the global feature parameter ${\hat{\beta }}_{t}$ in the inference process. We adopted the method of gradient descent for optimizing ${\hat{\beta }}_{t}$.

In Equation (16), the $\hat{\beta }$-related section is

(20) $$F\left(\hat{\beta }\right)\propto \prod_{t=1}^{T}\prod_{f=1}^{F+1}e^{-\frac{{\left(\beta_{tf}-\mu \right)}^{2}}{2\sigma^{2}}}\cdot \prod_{d=1}^{D}\frac{\Gamma \left(\sum_{t=1}^{T}\alpha_{dt}\Lambda_{dt}\right)}{\Gamma \left(\sum_{t=1}^{T}\alpha_{dt}\Lambda_{dt}+N_{d}\right)}\prod_{t=1}^{T}\frac{\Gamma \left(\alpha_{dt}\Lambda_{dt}+N_{dt}\Lambda_{dt}\right)}{\Gamma \left(\alpha_{dt}\Lambda_{dt}\right)}$$

Based on the logarithm of Equation (20), we take the derivative with respect to global feature parameter $\beta_{tf}$ and adjust it to zero. The updated equation of $\beta_{tf}$ is

(21) $$\begin{array}{cc} \beta_{tf}^{new}=\sigma^{2}\sum_{d=1}^{D}y_{df}\alpha_{dt}^{new}\Lambda_{dt}\{ & \Psi \left(\sum_{t=1}^{T}\alpha_{dt}^{new}\Lambda_{dt}\right)-\Psi \left(\sum_{t=1}^{T}\alpha_{dt}^{new}\Lambda_{dt}+N_{d}\right)\\ & +\Psi \left(\alpha_{dt}^{new}\Lambda_{dt}+N_{dt}\Lambda_{dt}\right)-\Psi \left(\alpha_{dt}^{new}\Lambda_{dt}\right)\}+\;\mu \\ t\in T=\{1,\ldots ,T\}\; & f\in F^{\prime }=\left\{1,\ldots ,F,F+1\right\} \end{array}$$

Finally, $\alpha_{dt}^{new}$ is updated by Equation (18).

#### 2.4.3. The Collapsed Gibbs Sampling Algorithm of the Dirichlet–Multinomial Regression Latent Dirichlet Allocation

To determine the initial state of the Markov chain, we initiate the hidden topic number $t_{dn}$ of each sample $x_{dn}$ first; then, we utilize the predictive probability of hidden variable $t_{dn}$ from Equation (17) as the state transition probability of the Markov chain. In the process of Gibbs sampling, the topic number $t_{dn}$ of each sample $x_{dn}$ is updated, and the hyper-parameter $\alpha_{d}={\left\{\alpha_{dt}\Lambda_{dt}\right\}}_{t=1}^{T}$ is also updated by Equation (18). Finally, the global feature parameter $\beta_{tf}$ is updated by Equation (21).

After several iterations in the burn-in time, the Markov chain is attracted to objective distribution, and then the posterior distribution $p\left(\beta ,T|W,\mu ,\sigma^{2},\alpha ,\lambda \right)$ is estimated. The posterior estimation of the local topic weight $\pi_{d}={\left\{\pi_{dt}\Lambda_{dt}\right\}}_{t=1}^{T}$ and topic–word multinomial distribution parameter is

(22) $${\hat{\pi }}_{dt}=\frac{\alpha_{dt}\Lambda_{dt}+E\left[N_{dt}\Lambda_{dt}\right]}{\sum_{t=1}^{T}\left(\alpha_{dt}\Lambda_{dt}+E\left[N_{dt}\Lambda_{dt}\right]\right)}$$

(23) $${\hat{\theta }}_{tw}=\frac{\lambda_{w}+E\left[N_{tw}\right]}{\sum_{w=1}^{W}\left(\lambda_{w}+E\left[N_{tw}\right]\right)}$$

#### 2.4.4. The Collapsed Variable Bayesian Inference Algorithm of the Dirichlet–Multinomial Regression Latent Dirichlet Allocation

The whole variational objective function before being collapsed is

(24) $$\begin{array}{ll} F\left(\eta \right) & =E_{q}\left[\log p\left(\beta ,\pi ,\theta ,T,W\right)\right]-E_{q}\left[\log q\left(\beta ,\pi ,\theta ,T|\eta \right)\right]\\ & =E_{q}\left[\log p\left(T,W\right)\right]-E_{q}\left[\log q\left(T|\eta \right)\right]\\ & =\mathrm{KL}\left(q\left(T|\eta \right)||p\left(T,W\right)\right) \end{array}$$

After margining the model parameters (π,θ), the objective function is

(25) $$\begin{array}{ll} F= & E_{q\left(t_{dn}\right)}\left[E_{q\left(T^{\left(\backslash dn\right)}\right)}\left[\log p\left(t_{dn},w_{dn}|T^{\left(\backslash dn\right)},W^{\left(\backslash dn\right)}\right)\right]\right]\\ & \;-E_{q\left(t_{dn}\right)}\left[\log q\left(t_{dn}\right)\right]+\;Const_{q\left(t_{dn}\right)} \end{array}$$

where $Const_{q\left(t_{dn}\right)}$ represents the unrelated item with variational distribution $q\left(t_{dn}\right)$. There are two kinds of construction below:

(26) $$F=\mathrm{KL}\left(q\left(t_{dn}\right)||\exp\left\{E_{q\left(T^{\left(\backslash dn\right)}\right)}\left[\log p\left(t_{dn},w_{dn}|T^{\left(\backslash dn\right)},W^{\left(\backslash dn\right)}\right)\right]\right\}\right)+\;Const_{q\left(t_{dn}\right)}$$

(27) $$F\geq \mathrm{KL}\left(q\left(t_{dn}\right)||E_{q\left(T^{\left(\backslash dn\right)}\right)}\left[p\left(t_{dn},w_{dn}|T^{\left(\backslash dn\right)},W^{\left(\backslash dn\right)}\right)\right]\right)+\;Const_{q\left(t_{dn}\right)}$$

In Equation (26), the updating equation of optimal variational parameter $\eta_{dnt}^{*}$ by a CVB algorithm is

(28) $$\begin{array}{ll} \eta_{dnt}^{*}= & q_{\mathrm{CVB}}^{*}\left(t_{dn}=t\right)\approx \exp\left\{E_{q\left(T^{\left(\backslash dn\right)}\right)}\left[\log\left(\alpha_{dt}+N_{dt}^{\left(\backslash dn\right)}\right)\Lambda_{dt}\right]\right\}\\ & +\exp\left\{E_{q\left(T^{\left(\backslash dn\right)}\right)}\left[\log\left(\lambda_{w}+N_{tw}^{\left(\backslash dn\right)}\right)\right]\right\}-\exp\left\{E_{q\left(T^{\left(\backslash dn\right)}\right)}\left[\log\left(\sum_{w=1}^{W}\lambda_{w}+N_{t}^{\left(\backslash dn\right)}\right)\right]\right\} \end{array}$$

Each expectation of the above equation is

(29) $$E_{q\left(T^{\left(\backslash dn\right)}\right)}\left[\log\left(\alpha_{dt}+N_{dt}^{\left(\backslash dw\right)}\right)\Lambda_{dt}\right]=\log\left(\alpha_{dt}\Lambda_{dt}+\mu_{N_{dt}^{\left(\backslash dw\right)}}\right)-\frac{\sigma_{N_{dt}^{\left(\backslash dw\right)}}^{2}}{2{\left(\alpha_{dt}\Lambda_{dt}+\mu_{N_{dt}^{\left(\backslash dw\right)}}\right)}^{2}}$$

(30) $$\left\{ \begin{array}{l} \mu_{N_{dt}^{\left(\backslash dw\right)}}=E_{q\left(T^{\left(\backslash dn\right)}\right)}\left[N_{dt}^{\left(\backslash dw\right)}\Lambda_{dt}\right]=\sum_{w=1}^{W}\left(N_{dw}-1\right)\eta_{dwt}\Lambda_{dt}\\ \sigma_{N_{dt}^{\left(\backslash dw\right)}}^{2}\;=V_{q\left(T^{\left(\backslash dn\right)}\right)}\left[N_{dt}^{\left(\backslash dw\right)}\Lambda_{dt}\right]=\;\sum_{w=1}^{W}\left(N_{dw}-1\right)\eta_{dwt}\Lambda_{dt}\left(1-\sum_{w=1}^{W}\left(N_{dw}-1\right)\eta_{dwt}\Lambda_{dt}\right) \end{array}\right.$$

(31) $$E_{q\left(T^{\left(\backslash dn\right)}\right)}\left[\log\left(\lambda_{w}+N_{tw}^{\left(\backslash dw\right)}\right)\right]=\log\left(\lambda_{w}+\mu_{N_{tw}^{\left(\backslash dw\right)}}\right)-\frac{\sigma_{N_{tw}^{\left(\backslash dw\right)}}^{2}}{2{\left(\lambda_{w}+\mu_{N_{tw}^{\left(\backslash dw\right)}}\right)}^{2}}$$

(32) $$\left\{ \begin{array}{l} \mu_{N_{tw}^{\left(\backslash dw\right)}}=E_{q\left(T^{\left(\backslash dw\right)}\right)}\left[N_{tw}^{\left(\backslash dw\right)}\right]=\sum_{d=1}^{D}\left(N_{dw}-1\right)\eta_{dwt}\Lambda_{dt}\\ \sigma_{N_{tw}^{\left(\backslash dw\right)}}^{2}\;=V_{q\left(T^{\left(\backslash dw\right)}\right)}\left[N_{tw}^{\left(\backslash dw\right)}\right]=\;\sum_{d=1}^{D}\left(N_{dw}-1\right)\eta_{dwt}\Lambda_{dt}\left(1-\left(N_{dw}-1\right)\eta_{dwt}\right)\Lambda_{dt} \end{array}\right.$$

(33) $$E_{q\left(T^{\left(\backslash dn\right)}\right)}\left[\log\left(\sum_{w=1}^{W}\lambda_{w}+N_{t}^{\left(\backslash dw\right)}\right)\right]=\log\left(\sum_{w=1}^{W}\lambda_{w}+\mu_{N_{t}^{\left(\backslash dw\right)}}\right)-\frac{\sigma_{N_{t}^{\left(\backslash dw\right)}}^{2}}{2{\left(\sum_{w=1}^{W}\lambda_{w}+\mu_{N_{t}^{\left(\backslash dw\right)}}\right)}^{2}}$$

(34) $$\left\{ \begin{array}{l} \mu_{N_{t}^{\left(\backslash dw\right)}}=E_{q\left(T^{\left(\backslash dw\right)}\right)}\left[N_{t}^{\left(\backslash dw\right)}\right]=\sum_{d=1}^{D}\sum_{w=1}^{W}\left(N_{dw}-1\right)\eta_{dwt}\Lambda_{dt}\\ \sigma_{N_{t}^{\left(\backslash dw\right)}}^{2}\;=V_{q\left(T^{\left(\backslash dw\right)}\right)}\left[N_{t}^{\left(\backslash dw\right)}\right]=\;\sum_{d=1}^{D}\sum_{w=1}^{W}\left(N_{dw}-1\right)\eta_{dwt}\Lambda_{dt}\left(1-\left(N_{dw}-1\right)\eta_{dwt}\Lambda_{dt}\right) \end{array}\right.$$

In Equation (27), the updating equation of the optimal variational parameter $\eta_{dnt}^{*}$ by CVB0 algorithm is

(35) $$\eta_{dnt}^{*}=q_{\mathrm{CVB}0}^{*}\left(t_{dn}=t\right)\approx \left(\alpha_{dt}\Lambda_{dt}+E_{q\left(T^{\left(\backslash dn\right)}\right)}\left[N_{dt}^{\left(\backslash dn\right)}\Lambda_{dt}\right]\right)\frac{\lambda_{w}+E_{q\left(T^{\left(\backslash dn\right)}\right)}\left[N_{tw}^{\left(\backslash dn\right)}\right]}{\sum_{w=1}^{W}\lambda_{w}+E_{q\left(T^{\left(\backslash dn\right)}\right)}\left[N_{t}^{\left(\backslash dn\right)}\right]}$$

The plenitude statistic of samples in Equation (35) are $N_{dt}^{\left(\backslash dn\right)}$,$N_{tw}^{\left(\backslash dn\right)}$, and $N_{t}^{\left(\backslash dn\right)}$, and their expectation under variational distribution $q\left(T^{\left(\backslash dn\right)}\right)$ is

(36) $$E_{q\left(T^{\left(\backslash dn\right)}\right)}\left[N_{dt}^{\left(\backslash dn\right)}\Lambda_{dt}\right]=\sum_{i=1,i\neq n}^{N_{d}}I\left(t_{dn}=t\right)\eta_{dit}\Lambda_{dt}$$

(37) $$E_{q\left(T^{\left(\backslash dn\right)}\right)}\left[N_{tw}^{\left(\backslash dn\right)}\right]=\sum_{d=1}^{D}\sum_{i=1,i\neq n}^{N_{d}}I\left(t_{dn}=t\right)I\left(w_{dn}=w\right)\eta_{dit}\Lambda_{dt}$$

(38) $$E_{q\left(T^{\left(\backslash dn\right)}\right)}\left[N_{t}^{\left(\backslash dn\right)}\right]=\sum_{d=1}^{D}\sum_{i=1,i\neq n}^{N_{d}}N_{di}\eta_{dit}\Lambda_{dt}$$

The \dn in the above equation can be adapted to \dw, because the bag of words dw not only shares a similar word number $w_{dn}=w$, but also shares the same topic number $t_{dn}=t$. Then, optimal variational distribution $\eta_{dnt}^{*}$ can be adapted to $\eta_{dwt}^{*}$.

(39) $$\gamma_{dt}=E_{q\left(T^{\left(\backslash dw\right)}\right)}\left[N_{dt}^{\left(\backslash dw\right)}\Lambda_{dt}\right]=\sum_{w=1}^{W}\left(N_{dw}-1\right)\eta_{dwt}\Lambda_{dt}$$

(40) $$\mu_{tw}=E_{q\left(T^{\left(\backslash dw\right)}\right)}\left[N_{tw}^{\left(\backslash dw\right)}\right]=\sum_{d=1}^{D}\left(N_{dw}-1\right)\eta_{dwt}\Lambda_{dt}$$

(41) $$\mu_{t}=E_{q\left(T^{\left(\backslash dw\right)}\right)}\left[N_{t}^{\left(\backslash dw\right)}\right]=\sum_{d=1}^{D}\sum_{w=1}^{W}\left(N_{dw}-1\right)\eta_{dwt}\Lambda_{dt}$$

The inference equation difference between CVB and CVB0 shows that CVB only retains the zero-order information of the Taylor expansion; however, CVB0 is the re-collapse of a hidden variable space based on Jensen inequality. Therefore, CVB0 is much more precise than CVB. The corresponding algorithm of CVB and CVB0 are shown in Table 1 and Table 2 respectively.

## 3. Materials and Results

This section provides a concise and precise description of the experimental results, their interpretation, and the experimental conclusions that can be drawn.

### 3.1. Dataset

In this paper, the validity and accuracy of proposed models are tested on the S.cerevisiae (S.C) dataset, which is introduced in reference [12]. This dataset includes several aspects of the yeast genome, such as sequence statistics, phenotype, expression, secondary structure, and homology. Meanwhile, two kinds of function annotation standard, including FunCat and GO, are used to annotate gene function. Due the universality of GO, the dataset depends on the GO that is adopted in our experiments. As described in Section 2.1, the construction of the BoW is based on amino acid composition, so we mainly use one of datasets that depends on the sequence statistics. In addition, we construct a dataset named S.C-CC from S.C, which only includes the GO terms belonging to the cellular component (CC). Therefore, there are fewer GO terms in the S.C-CC dataset when compared with the S.C dataset, and both of them are used in our experiments for investigating the influence of different label numbers on prediction performance. The statistics of the S.C and S.C-CC dataset is shown in Table 3. In this set, F denotes the number of GO terms, D denotes the number of genes, and W denotes the size of the vocabulary.

As shown in Table 3, there are 1692 genes and 4133 function labels in the S.C dataset; in the S.C-CC dataset, there are 1692 genes and 547 function labels. Due to the large number of GO terms in the gene function dataset, we utilized a Boolean matrix decomposition (BMD) method to reduce the dimensionality of the function labels. BMD is a kind of label space dimension reduction (LSDR) method [19], which addresses the multi-label classification problem with many labels. LSDR approaches use a compression step to transform the original high dimension label space into a lower dimension label space, and then multi-label classifiers are trained on a dataset with fewer labels, which can reduce the computation burden of the classifier. The existing studies about LSDR show that LSDR approaches are useful for optimizing the running time and accuracy of multi-label classification. In our BMD process, original label matrix $Y\in {\left\{0,1\right\}}^{D\times F}$ (D denotes the number of genes, and F denotes the number of features) is decomposed into the product of two matrices, $C\in {\left\{0,1\right\}}^{D\times L}$ (L denotes the number of labels) and $B\in {\left\{0,1\right\}}^{L\times F}$, where Y=C∘B (∘ denotes Boolean product) is satisfied. We also called it exact BMD and adopted this algorithm, which is proposed in reference [20]. Compared with other LSDR algorithms, an exact BMD can retain the interpretability of low dimension label space and restore the low dimension-predicted label matrix to the original label matrix by matrix B. At last, the number of function labels is reduced into a smaller dimension, and L denotes the number of GO terms after label space dimension reducing. Then DMR-LLDA actually needs to process 1358 GO terms of the S.C dataset and 319 GO terms of the S.C-CC dataset. Nonetheless, the lower dimensional label space can be recovered by a Boolean product after predicting, so we still get the whole function labels sets in the prediction results.

DMR-LLDA’s advantage here over LLDA lies in the introduction of extra features. In the S.C and S.C-CC dataset, there are six extra gene features for each gene, including the molecular weight of the gene, the isoelectric point, the average coefficients of hydrophilic, the number of exons, the adaptability index of the codon, the number of motifs, and the open reading frame (ORF) number of chromosomes. The statistics of extra features are shown in Table 4.

As the max word length of the S.C dataset is two amino acid alphabets (G,A,V,L,I,F,P, Y,S,C,M,N,Q,T,D,E,K,R,H,W), a human dataset constructed by ourselves is adopted to evaluate the impact on the topic model performance of word length. This human dataset is constructed in a similar way as in reference [13]. In addition, we also constructed two human datasets for different word lengths, where the max word length of the Human1 dataset is two amino acid alphabets, and that of the Human2 dataset is three amino acid alphabets. For the Human2 dataset, the original number of words is 8400, but we filtered several words that have a high frequency. Then, the statistic of the S.C and S.C-CC dataset is shown in Table 5.

### 3.2. Parameter Settings and Evaluation Criterias

The DMR-LLDA learning framework involves four different parameters: μ, $\sigma^{2}$, α, and λ. The α and λ are the parameters of the two-Dirichlet distribution, where the larger the value of λ, the more balanced the probability of a word in a topic. The setting of the λ value has been discussed in reference [13]. Nonetheless, the value of α is optimized by a protein feature, so its initial value does not have a big effect on model performance. According to the experience, we set α=50/T as the initial value, and set λ=200/W, with T=L. In addition, μ and $\sigma^{2}$ are respectively the mean and variance of normal distribution, obeyed by feature weighted parameter β, and we set $\mu =0,\sigma^{2}=1$.

In the Gibbs sampling process of model training, we set the number of the Markov chain as 1 and the maximum number of iterations is 2000 times, where the number of iterations of burn-in time is set to 1000. We record the state space at intervals of 50 times on the converged Markov chain, and 20 times per record is conducted. In the process of model predicting, we set the number of iterations as 1000 times. After 500 iterations for the burn-in time, we record the state space at intervals of 50 times. In the variable Bayesian inferring process of model training, we initialize the global variable parameter $\mu_{tw}$ through random number s and hyper-parameter $\lambda_{w}$: $\mu_{tw}=\lambda_{w}+(s*\lambda_{w})/10$; in each local variable inference, we set the converged threshold as 0.00001, and the maximum number of times of the local variable inference as 100. The number of global scanning iterations is 1000.

The five-fold cross validation is conducted to measure and compare the performance of DMR-LLDA and the comparative algorithms. Five representative multi-label learning evaluation criteria are used in this paper, including hamming loss (HL), average precision (AP), one error, and micro-averaged and macro-averaged F1 scores (Micro-F1 and Macro-F1). In addition, three kinds of areas under a precision–recall curve are also used, including $\bar{AUPRC}$, $AU(\bar{PRC})$, and $\bar{AUPRCw}$, which is proposed in reference [12]. Finally, we repeat the random partition and evaluation in five independent rounds, and report the average results.

### 3.3. The Impact of Word Length on Model Performance

**Figure 4:**
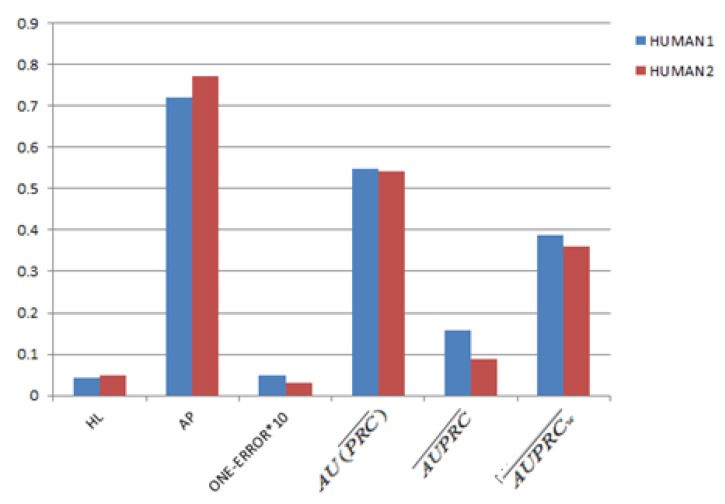
The comparisons between the Human1 and Human2 datasets. Define terms if necessary.

Firstly, the performance comparison of the LLDA model between the Human1 and Human2 datasets is shown in Figure 4. As shown in Figure 4, we find that the value of $\bar{AUPRC}$ and $\bar{AUPRCw}$ in Human1 is higher than that in Human2; the value of the AP on Human1 is lower than that of Human2; and the value of one error, HL, and $AU\bar{\left(PRC\right)}$ is almost equal to that of Human1 and Human2. These results show that the classification performance of the LLDA on Human1 and Human2 is almost the same, which reveals that the larger word space might not obtain a better classifying performance.

Moreover, related studies suggested that a word length of more than four amino acid alphabets would not improve the classification accuracy, and would only increase the complexity of computation [21]. Therefore, in the following experiments, we only adopt the S.C and S.C-CC datasets whose word length is two amino acid alphabets.

### 3.4. Gene Function Prediction with Cross Validation

**Figure 5:**
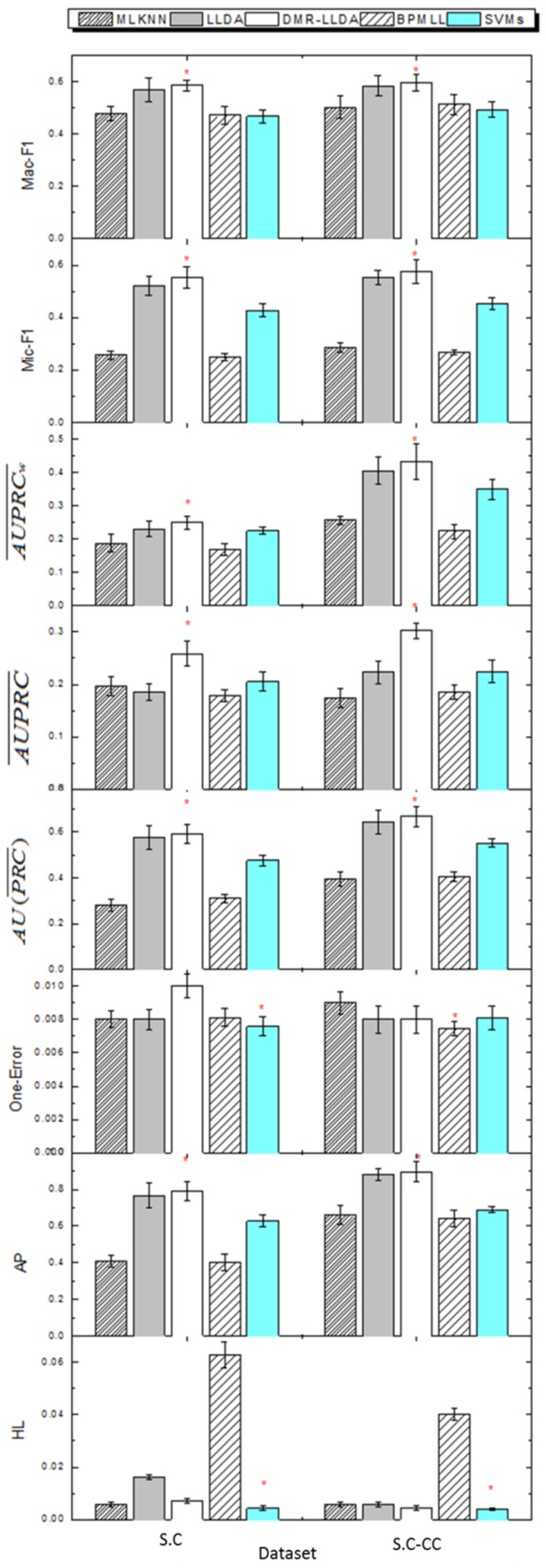
The comparisons between DMR-LLDA, LLDA, back propagation for multi-label learning (BPMLL), support vector machines (SVMs), and multi-label *k*-nearest neighbor (MLKNN) in two datasets. The red asterisk represents the best results in each dataset.

In addition to LLDA, we also adopted three widely adopted methods: multi-label *k*-nearest neighbor (MLKNN) [22], back propagation for multi-label learning (BPMLL) [23], and support vector machines (SVMs) for performance comparison. MLKNN and BPMLL are two representative multi-label classifiers, and can be performed by an open source tool called Mulan [24]. SVMs adopt a “one-versus-all” scheme, which trains each label by a binary SVM independently and is implemented using the LibLinear software package [25]. These five models are trained and used to predict with the S.C and S.C-CC datasets. Figure 5 shows the HL, AP, one error, Micro-F1, Macro-F1, $AU(\bar{PRC})$, $\bar{AUPRC}$, and $\bar{AUPRCw}$ values of all models in the two datasets, respectively. For AP, Micro-F1, Macro-F1, $AU(\bar{PRC})$, $\bar{AUPRC}$, and $\bar{AUPRCw}$, the larger the value, the better the performance. Conversely, for HL and one error, the smaller the value, the better the performance. The red asterisk of Figure 5 represents the best results in each dataset. It is worth noting that the experimental results of this section are obtained by a CGS inference algorithm.

As shown in Figure 5, DMR-LLDA can achieve better results in almost all evaluation criteria for the two datasets. The concrete analysis is introduced as follows.

For the S.C dataset, the DMR-LLDA achieves the best performance for AP, $AU(\bar{PRC})$, $\bar{AUPRC}$, $\bar{AUPRCw}$, Micro-F1, and Macro-F1. For example, with AP, the DMR-LLDA achieves 94%, 3.3%, 96%, and 26% improvements over MLKNN, LLDA, BPMLL, and SVMs, respectively. With $AU(\bar{PRC})$, the DMR-LLDA achieves 109%, 2.3%, 89%, and 24% improvements over MLKNN, LLDA, BPMLL, and SVMs, respectively. For $\bar{AUPRC}$, the DMR-LLDA achieves 31%, 39%, 44%, and 25% improvements over MLKNN, LLDA, BPMLL, and SVMs, respectively. For $\bar{AUPRCw}$, the DMR-LLDA achieves 33%, 8.1%, 48%, and 10% improvements over MLKNN, LLDA, BPMLL, and SVMs, respectively. With Micro-F1, the DMR-LLDA achieves 116%, 6.1%, 123%, and 29% improvements over MLKNN, LLDA, BPMLL, and SVMs, respectively. On Macro-F1, DMR-LLDA achieves 22%, 2.9%, 24%, and 25% improvements over MLKNN, LLDA, BPMLL, and SVMs, respectively. Nevertheless, for one error and HL, SVMs get better results than the DMR-LLDA.

For the S.C-CC dataset, the DMR-LLDA obtains a better performance in terms of AP, $AU(\bar{PRC})$, $\bar{AUPRC}$, $\bar{AUPRCw}$, Micro-F1, and Macro-F1. For AP, the DMR-LLDA achieves 36%, 1.7%, 39%, and 30% improvements over MLKNN, LLDA, BPMLL, and SVMs, respectively. For $AU(\bar{PRC})$, the DMR-LLDA achieves 68%, 4%, 64%, and 20% improvements over MLKNN, LLDA, BPMLL, and SVMs, respectively. For $\bar{AUPRC}$, the DMR-LLDA achieves 73%, 35%, 62%, and 34% improvements over MLKNN, LLDA, BPMLL, and SVMs, respectively. For $\bar{AUPRCw}$, the DMR-LLDA achieves 67%, 6.4%, 92%, and 23% improvements over MLKNN, LLDA, BPMLL, and SVMs, respectively. For Micro-F1, the DMR-LLDA achieves 101%, 4.1%, 114%, and 26% improvements over MLKNN, LLDA, BPMLL, and SVMs, respectively. For Macro-F1, the DMR-LLDA achieves 18%, 1.8%, 16%, and 20% improvements over MLKNN, LLDA, BPMLL, and SVMs, respectively. Nevertheless, for one error, BPMLL gets better results than the DMR-LLDA; for HL, SVMs gets better results than the DMR-LLDA.

For both of the datasets, we can find that the improvements on $\bar{AUPRC}$ are more significant than $AU(\bar{PRC})$ and $\bar{AUPRCw}$, which indicates that the DMR-LLDA has a stronger effect on improving the overall accuracy of gene function prediction without respect to label weights. In the comparisons of the S.C and S.C-CC datasets, we find that the values of AP, $AU\bar{\left(PRC\right)}$, $\bar{AUPRC}$, and $\bar{AUPRCw}$ in the S.C dataset is lower than in the S.C-CC dataset, and the value of one error and HL in the S.C is higher than in the S.C-CC dataset. This is due to the same word space and different label number between these two datasets. The fewer labels of the S.C-CC dataset can promote a higher classifying performance.

Above all, these results indicate that the DMR-LLDA can further improve the accuracy of gene function prediction by introducing the DMR framework into the LLDA model, which optimizes the hyper-parameters of the topic weight. Meanwhile, the DMR-LLDA has an apparent advantage in improving the overall prediction accuracy.

### 3.5. The Impact on Prior Parameters of Feature Variables

In the DMR-LLDA model, the introduction of gene features is realized by feature weight parameter β. Then the operation on topic parameter vector $\beta_{t}$ and feature vector $y_{d}$ are reflected in Dirichlet hyper parameter $\alpha_{d}$. Table 6 shows the impact of different feature values on prior parameters $\alpha_{d}$.

For the LLDA, the hyper-parameter value is set as a fixed value. However, Table 6 shows that only the different values on mol_wt, theo_pI, hydro, and Cai make a significant difference of hyper-parameter value in the DMR-LLDA, which is also the main way for gene features to impact label allocation.

### 3.6. The Comparison Results of Inference Algorithms

**Figure 6:**
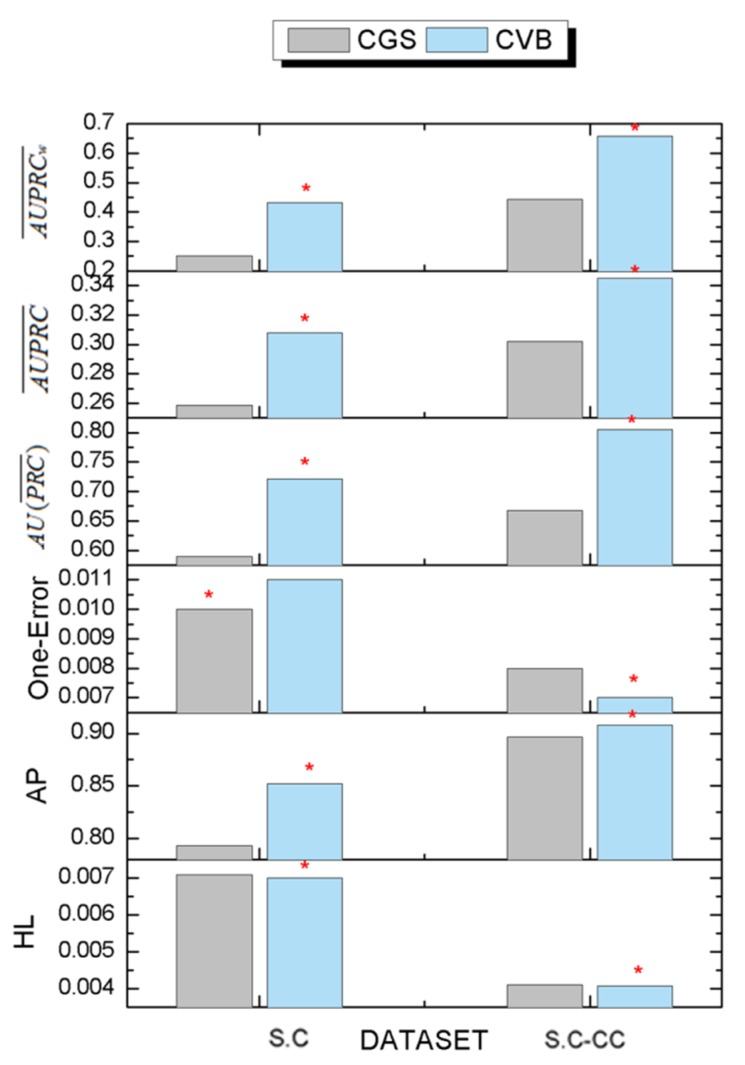
The comparison results with CVB0 and CGS. The red asterisk represents the best results in each dataset.

We designed three kinds of inference algorithm for the DMR-LLDA, including CGS, CVB, and CVB0. This section compares CGS with CVB0 in the S.C dataset. The experimental results are shown in Figure 6. As shown in Figure 6, the overall performance of CVB0 is better than the performance of CGS. Concrete analysis is represented as follows.

For the S.C dataset, CVB0 achieves the best results in AP, $\bar{AUPRC}$, $AU(\bar{PRC})$, and $\bar{AUPRCw}$, and achieves almost similar results in HL. However, CVB0 has a worse value in one error.

For the S.C-CC dataset, CVB0 achieves the best results in AP, one error, $\bar{AUPRC}$, $AU(\bar{PRC})$, and $\bar{AUPRCw}$, and achieves almost similar results in HL. The above results demonstrate the validity of the designed inference algorithms for the DMR-LLDA. Meanwhile, the experimental results indicate that the CVB0 inference algorithm can obtain more precise prediction results by the re-collapse of hidden variable space based on Jensen inequality.

Above all, due to the lack of prior knowledge, the prior distributions of the Bayesian model are usually set for convenience. Meanwhile, the parameters of prior distribution are also set as a fixed value based on experience, which makes the inaccurate estimation of posterior distributions. In our DMR-LLDA model, the gene feature information is introduced into the LLDA as the prior knowledge by the DMR framework. The hyper-parameter of the prior distribution is updated in the inference process rather than by a fixed constant, which can improve the estimation precision of posterior distributions, so as to improve the accuracy of gene function prediction.

## 4. Conclusions

In this paper, we introduce multiple types of features into gene function prediction based on a multi-label surprised topic model, and propose a multi-label supervised topic model conditioned on arbitrary features named the DMR-LLDA. By applying an exponential a priori constructed previously with weighted features on the hyper-parameters of gene-topic (or label) distribution, this model can utilize the observed features of each gene in multi-label topic modeling. Furthermore, three learning algorithms are designed for this model, including CGS, CVB inference, and CVB0 inference. The predictive performance of this model is measured by the AP, one error, Hamming loss, $\bar{AUPRC}$, $AU(\bar{PRC})$, $\bar{AUPRCw}$, Micro-F1, and Macro-F1. Experiments on a standard dataset show that the DMR-LLDA is superior to the LLDA, MLKNN, BPMLL, and SVM models. Meanwhile, experimental results show that the DMR-LLDA can get a much more accurate estimation of posterior distribution, due to using the gene feature information in addition to the amino acid sequence.

## Figures and Tables

**Table 1:** Collapsed variable Bayesian (CVB) algorithm of DMR-LLDA.

	**CVB algorithm of DMR-LLDA**
1	Initialize global variational parameters
2	While the number of iterations $r<r_{\max}$ or F is not converged do
3	For d=1:D do
4	Initialize local variational parameters to constant
5	Repeat (the local variational inference of gene d)
6	$\eta_{dwt}^{\left(r\right)}\propto \frac{\left(\Lambda_{dt}\alpha_{dt}+\mu_{N_{dt}}^{\left(r-1\right)}\right)\;\left(\lambda_{w}+\mu_{N_{tw}}^{\left(r-1\right)}\right)}{\left(\sum_{w=1}^{W}\lambda_{w}+\mu_{N_{t}}^{\left(r-1\right)}\right)}e^{-\frac{{\left(\sigma_{N_{dt}}^{\left(r-1\right)}\right)}^{2}}{2{\left(\alpha_{t}+\mu_{N_{dt}}^{\left(r-1\right)}\right)}^{2}}}e^{-\frac{{\left(\sigma_{N_{tw}}^{\left(r-1\right)}\right)}^{2}}{2{\left(\lambda_{w}+\mu_{N_{tw}}^{\left(r-1\right)}\right)}^{2}}}e^{-\frac{{\left(\sigma_{N_{t}}^{\left(r-1\right)}\right)}^{2}}{2{\left(\sum_{w=1}^{W}\lambda_{w}+\mu_{N_{t}}^{\left(r-1\right)}\right)}^{2}}}$
7	Update $\mu_{N_{dt}}^{\left(r\right)}$ and $\sigma_{N_{dt}}^{\left(r\right)}$ by Equations (29)~(30)
8	$\alpha_{dt}^{\left(r\right)}=\exp\left({\hat{y}}_{d}{\hat{\beta }}_{t}^{\left(r-1\right)}\right)$
9	Until $\gamma_{dt}^{\left(r\right)}$ is converged: $\left(1/N_{d}\right)\sum_{t=1}^{T}\left|\gamma_{dt}^{\left(r\right)}-\gamma_{dt}^{\left(r-1\right)}\right|<0.00001$
10	End For
11	$\mu_{N_{tw}}^{\left(r\right)}$, $\sigma_{N_{tw}}^{\left(r\right)}$, $\mu_{N_{t}}^{\left(r\right)}$ and $\sigma_{N_{t}}^{\left(r\right)}$ by Equations (31)~(34)
12	Update $\beta_{tf}^{\left(r\right)}$ by Equation (21)
13	End while

**Table 2:** Zero-order variational Bayesian (CVB0) algorithm of DMR-LLDA.

	**CVB0 algorithm of DMR-LLDA**
1	Initialize global variational parameters
2	While the number of iterations $r<r_{\max}$ or F is not converged do
3	For d∈D do
4	Initialize local variational parameters to constant
5	Repeat: (the local variational inference of gene d)
6	$\eta_{dwt}^{\left(r\right)}\propto \left(\alpha_{dt}\Lambda_{dt}+\gamma_{dt}^{\left(r-1\right)}\right)\frac{\lambda_{w}+\mu_{tw}^{\left(r-1\right)}}{\sum_{w=1}^{W}\lambda_{w}+\mu_{t}^{\left(r-1\right)}}$
7	$\gamma_{dt}^{\left(r\right)}=\sum_{w=1}^{W}\left(N_{dw}-1\right)\eta_{dwt}^{\left(r\right)}$
8	$\alpha_{dt}^{\left(r\right)}=\exp\left({\hat{y}}_{d}{\hat{\beta }}_{t}^{\left(r-1\right)}\right)$
9	Until $\gamma_{dt}^{\left(r\right)}$ is converged: $\left(1/N_{d}\right)\sum_{t=1}^{T}\left|\gamma_{dt}^{\left(r\right)}-\gamma_{dt}^{\left(r-1\right)}\right|<0.00001$
10	End For
11	$\mu_{tw}^{\left(r\right)}=\sum_{d=1}^{D}\left(N_{dw}-1\right)\eta_{dwt}^{\left(r\right)}$, $\mu_{t}^{\left(r\right)}=\sum_{d=1}^{D}\sum_{w=1}^{W}\left(N_{dw}-1\right)\;\eta_{dwt}^{\left(r\right)}$
12	Update $\beta_{tf}^{\left(r\right)}$ by Equation (21)
13	End while

**Table 3:** The statistics of the S.cerevisiae (S.C) and S.cerevisiae-cellular component (S.C-CC) datasets.

Dataset	D	W	F	L
S.C	1692	400	4133	1538
S.C-CC			547	319

**Table 4:** The statistics of extra features in the S.C dataset.

Feature Name	Notation	Type
molecular weight	mol_wt	Integer
isoelectric point	theo_pI	Real numbers
average coefficients of hydrophilic	hydro	Real numbers
number of exons	position	Integer
adaptability index of codon	Cai	Real numbers
number of motifs	motifs	Integer
ORF number of chromosomes	chromosome	Integer

ORF: Open reading frame

**Table 5:** The statistic of two human datasets.

Dataset	D	W	L
Human1	4962	5297	1477
Human2		400	

**Table 6:** The impact on prior parameters of feature variables.

$\alpha_{d}$	The words under topic when mol_wt = 49629.3, theo_pI = 8.96, hydro = 0.1, position = 1, Cai = 0.17, motifs = 2, chromosome = 16
1.88	GM IH LH VH LK IG GC IC AK VM FG AM LW IK VG VW FC IG FH GK
1.32	LM ST SM LT KM LL IM KL LF SL EM LP DM IT LK EF KT LE SK SP
0.79	GH VC AC KC GC AL GM LH AH AF AM VW AW GW EC KH TH GF AT GT
0.64	IL VG GE FM YK QW YM VW GP TL KT LW RP LQ IR FH NW NX FS PM
0.23	TT SV TV ST SW SQ TQ PT PV IV SP TM CT QT AV TP TC SC VV NV
$\alpha_{d}$	The words under topic when mol_wt = 85873.7, theo_pI = 9.74, hydro=0.664 position = 1, Cai = 0.1, motifs = 2, chromosome = 16
4.23	KR TF KE QS LW EW DM YF QT SM LX SF IN QW LR VL VS QG MC QC
3.77	LM SM LS RC DW EM LE QT LV EW FM QI RM NE DT IE FT AR QC GP
0.23	QM KR AP EF LF QR HP EC RE RF DS VE EW KF FE LT TL QV QC AR
0.11	CF PI ED QY GQ HN RI HD HI SN YQ TQ PW RH YL PQ PN SI QE RS
0.09	SW VF NW AC DF TW EQ LW EH MC DM AW PS GV VQ AQ ID TG RF VE
